# The controversial role of linoleic acid in cardiometabolic health: from molecular pathways to human studies

**DOI:** 10.3389/fnut.2025.1728865

**Published:** 2026-01-14

**Authors:** Loni Berkowitz, Paloma Araneda, Glenda Cofré, Daniela Sara, Mariano Olsen, Isadora Urzúa, Druso Pérez, Attilio Rigotti

**Affiliations:** 1 Centro de Nutrición Molecular y Enfermedades Crónicas, Escuela de Medicina, Pontificia Universidad Católica de Chile, Santiago, Chile; 2 Departamento de Nutrición, Diabetes y Metabolismo, Escuela de Medicina, Pontificia Universidad Católica de Chile, Santiago, Chile

**Keywords:** cardiometabolic health, cardiovascular diseases, dietary fats, linoleic acid, omega-6 fatty acids

## Abstract

Unhealthy diets are major contributors to the global burden of non-communicable diseases, particularly cardiovascular disease and metabolic syndrome, where dietary fat quality plays a critical role. Among dietary fats, linoleic acid (LA)—the predominant omega-6 polyunsaturated fatty acid—has been at the center of a long-standing and evolving controversy. Initially promoted for its cholesterol-lowering properties, LA later became the focus of debate due to hypotheses suggesting pro-inflammatory and oxidative effects, which led to conflicting interpretations of its metabolic impact and inconsistent dietary guidelines over time. This review traces the origins and progression of this controversy, examining how shifts in biochemical understanding, experimental design, and population dietary patterns have shaped current perspectives on LA and cardiometabolic health. By integrating evidence from biochemical, preclinical, and human studies, we clarify the mechanistic and clinical bases underlying LA’s actions and re-evaluate its role in lipid metabolism, inflammation, and glucose regulation. Overall, most human evidence supports beneficial associations between LA exposure and cardiometabolic outcomes, though heterogeneity across studies underscores the relevance of dietary context, genetic background, and metabolic status. Understanding how the controversy emerged and evolved is essential to refine current recommendations for dietary fat and disease prevention.

## 1 Introduction

Non-communicable diseases (NCDs) are currently major causes of morbidity and mortality representing a large proportion of the overall economic healthcare burden worldwide (1). Even worse, these diseases (mainly obesity, diabetes, cancer, as well as, cardiovascular, respiratory, and neurodegenerative conditions) are increasing in most world regions, currently representing ≈70% of all annual deaths (1). Specifically, cardiovascular diseases (CVDs) are the leading cause of death globally, accounting for at least 19 million lives each year (1). Metabolic Syndrome (MetS), a cluster of cardiometabolic risk factors, is linked to a two-fold higher risk of cardiovascular disease and a five-fold greater risk of type 2 diabetes (T2D) (2). Its global prevalence ranges from 12.5% to more than 40% in some Latin American countries (3, 4), with increasing burden particularly in youth and young adults, rising current and future healthcare and economic costs.

Central obesity is considered the key pathophysiological driver, promoting insulin resistance via adipose tissue dysfunction (5, 6). Excess free fatty acids and pro-inflammatory cytokines from visceral fat led to ectopic fat deposition, inflammation, and impaired insulin signaling. This cascade triggers hyperinsulinemia, atherogenic dyslipidemia, and endothelial dysfunction, thereby amplifying cardiometabolic risk (5–7).

Among the modifiable factors, an unhealthy diet is the most relevant risk factor for the development of MetS and NCDs. For instance, an aggregate of dietary subcomponents was more significantly associated with chronic disease burden than either physical inactivity or high body mass index in US population (8). Whereas unhealthy diets adversely affect human health, healthy nutrient and food intake can prevent various NCDs (9). In this context, the definition of a healthy diet is constantly evolving, reflecting our growing understanding of the roles of different foods, nutrients, and dietary combinations in health (10, 11). A clear example of this evolution are the recommendations for fat intake. For several decades, many dietary guidelines emphasized reducing total fat intake (12). Now, strong evidence supports that the type of fat (i.e., saturated, trans, monounsaturated and polyunsaturated fatty acids) is more relevant to cardiovascular health than total fat intake (13). In fact, the Mediterranean diet is considered the healthiest dietary patterns today (14), and it encourages moderate consumption of “healthy fats” provided by olive oil and fish (15, 16).

Overall, the current epidemiological situation calls out an urgent need for further study of the relationship between eating patterns, with particular emphasis in fat intake, and the prevention of MetS and other chronic diseases. Among dietary fats, linoleic acid (LA)—the most abundant omega-6 polyunsaturated fatty acid in the human diet—warrants particular attention (17). Thus, LA plays a significant role in overall fat intake. However, its impact on cardiometabolic health remains controversial, with studies suggesting both beneficial and potentially adverse effects depending on dietary context, dose, and metabolic state.

In this review, we explore the controversial role of linoleic acid in cardiometabolic health by examining its metabolism and bioactive derivatives, key molecular pathways, and evolving dietary recommendations in relation to current intake patterns. We address the debate over its protective versus detrimental effects by integrating evidence from biochemical, preclinical, observational, and intervention studies in humans.

## 2 Linoleic acid metabolism and its derivatives: the origin of the controversy

Accumulating evidence suggests that the health effects of dietary fats vary depending on both specific fatty acids and food sources (13). Dietary fatty acids are conventionally grouped by the presence or absence of carbon–carbon double bonds. Fatty acids with no double bonds are saturated (SFAs), those with one double bond are monounsaturated (MUFAs), and those with two or more double bonds are polyunsaturated (PUFAs). PUFAs can be further subdivided by the position of the first double bond at the third (*ω*-3) or sixth (ω-6) carbon from the methyl end of the hydrocarbon chain. While a high intake of SFA is associated with an increased risk of cardiometabolic diseases, evidence suggests that the intake of PUFAs has beneficial roles in human health (17–20).

**Figure 1:**
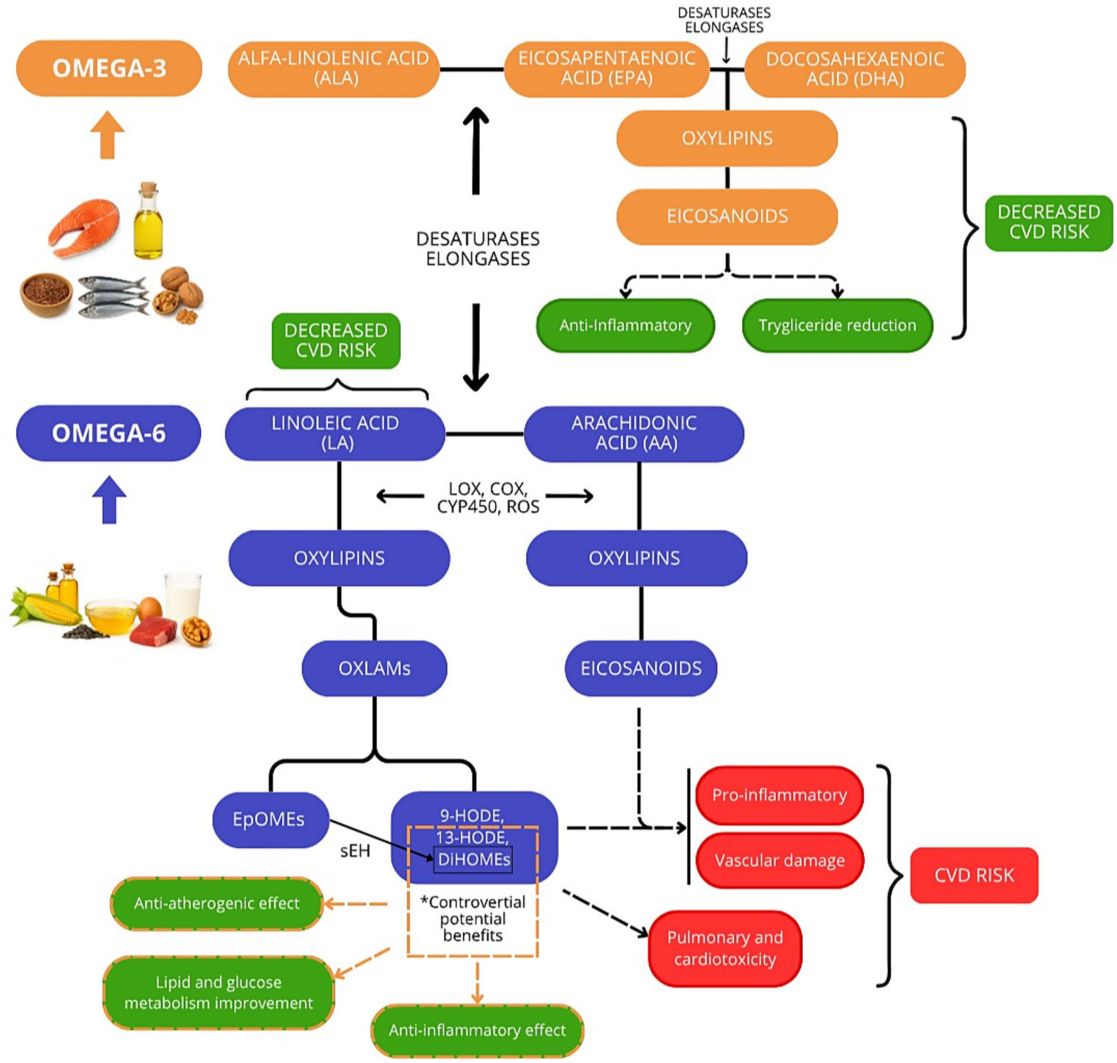
Metabolism and proposed pathophysiological roles of omega-6 and omega-3 fatty acids. Linoleic acid (LA, 18:2 n-6) and *α*-linolenic acid (ALA, 18:3 n-3) are the essential precursors of the *ω*-6 (blue) and ω-3 (orange) families, respectively. Through sequential desaturation and elongation steps—catalyzed by shared enzymes such as Δ6-desaturase and elongase—LA is converted to arachidonic acid (AA), whereas ALA gives rise to eicosapentaenoic acid (EPA) and docosahexaenoic acid (DHA). Both LA and ALA can also undergo direct enzymatic or non-enzymatic oxidation, generating diverse bioactive oxylipins. In the figure, green boxes indicate potential beneficial effects on cardiometabolic health, while red boxes denote those linked to increased cardiometabolic risk as reported in the literature.

In most diets, PUFAs present in the highest amounts are linoleic acid (LA, 18:2*ω*-6) and *α*-linolenic acid (ALA, 18:3ω-3) (21, 22). LA and ALA (the shortest-chained ω-6 and ω-3 fatty acids, respectively) are not synthesized by the human body and so are regarded as essential fatty acids (18). Because they are produced in plants, LA and ALA are mainly found in high proportions in oily foods of plant origin, such as many seeds, nuts, and plant oils (17). Endogenously, LA can be partially converted into several other *ω*-6 PUFAs including arachidonic acid (AA) (23) (Figure 1). These longer ω-6 PUFAs are also present in small amounts in foods such as eggs, poultry, and other meats. Similarly, ALA can to some extent be enzymatically converted into more complex *ω*-3 fatty acids, such as eicosapentaenoic acid (EPA), and docosahexaenoic acid (DHA; Figure 1). However, these conversions occur in low quantities in humans (24, 25), such that tissue and circulating levels of EPA and DHA are mostly determined by their direct dietary intake, mostly in fish and shellfish, with smaller amounts present in eggs, red meats and poultry (17, 26).

PUFAs are also precursors of bioactive metabolites, such as eicosanoids and docosanoids (Figure 1). In general, many eicosanoids derived from *ω*-6 PUFA, particularly those originating from arachidonic acid, exhibit pro-inflammatory and pro-thrombotic properties (e.g., prostaglandins and leukotrienes) (23); however, certain *ω*-6–derived eicosanoids, such as those derived from dihomo-*γ*-linolenic acid, exert anti-inflammatory effects (27). In contrast, eicosanoids and docosanoids derived from *ω*-3 PUFA are predominantly anti-inflammatory (e.g., protectins or resolvins) (23). Beyond these classical mediators, ω-3 and ω-6 PUFAs are major precursors of a broader family of oxidized lipid metabolites, collectively known as oxylipins. Oxylipins play critical roles in the regulation of inflammatory pathways, vascular homeostasis, and energy metabolism. These bioactive mediators arise through both enzymatic pathways, involving cyclooxygenases, lipoxygenases, and cytochrome P450 enzymes, as well as non-enzymatic mechanisms driven by oxidative stress (28).

The relative production of oxylipins and their impact on cardiometabolic health depends both on the fatty acid of origin and on the enzymatic pathways involved in their formation (29). While oxylipins derived from *ω*-3 fatty acids, such as EPA and DHA, generally exert anti-inflammatory and protective effects, those originating from *ω*-6 PUFAs tend to promote inflammation and oxidative stress (28, 29) (Figure 1).

Among the subset of oxylipins derived from ω-6 PUFAs, oxidized linoleic acid metabolites (OXLAMs) have attracted particular attention. OXLAMs such as 9-hydroxyoctadecadienoic acid (9-HODE), 13-hydroxyoctadecadienoic acid (13-HODE), epoxyoctadecenoic acid (EpOMEs) and their hydrolized metabolites, dihydroxyoctadecenoic acid (DiHOMEs), have been associated with different physiological effects (28). While 9-HODE has been linked to vascular injury, 13-HODE appears to exert protective roles in both atherosclerosis and metabolism. In vascular biology, 13-HODE has been associated with anti-atherogenic effects through the reduction of endothelial adhesion and stimulation of prostacyclin synthesis (30). In addition, it contributes to metabolic regulation by improving lipid and glucose homeostasis in metabolic syndrome and diabetes (31). These actions are further supported by anti-inflammatory properties, including the suppression of NF-κB signaling and modulation of T-cell IL-2 production (32). Meanwhile, although EpOMEs may initially serve protective roles, their conversion to DiHOMEs has largely been linked to cytotoxic pathways involved in pulmonary and cardiotoxicity, as well as inflammation. Nevertheless, at low physiological concentrations, 12,13-DiHOME has emerged as a beneficial lipokine, enhancing fatty acid uptake and oxidation in brown adipose tissue and skeletal muscle during cold exposure and exercise, lowering circulating triglycerides, improving lipid metabolism and insulin sensitivity, and contributing to vascular repair—without adverse cardiovascular effects (33, 34).

Given that mammals lack the enzymatic capacity to synthesize LA *de novo*, tissue levels of LA and its oxidized metabolites are likely influenced by dietary intake (35, 36). Importantly, synthesis of long-chain PUFAs and their derivatives from both dietary LA (*ω*-6 precursor) and ALA (ω-3 precursor) share the same enzymatic system and can compete for this biochemical pathway (23, 37). Based on this theoretical competition between *ω*-6 PUFAs and ω-3 PUFAs and the pro-inflammatory effects of eicosanoids and oxidized metabolites derived from ω-6 PUFAs, a group of scientists proposed the notion of lowering the dietary *ω*-6:ω-3 ratio, including recommendations to lower LA intake (38–42). In fact, this viewpoint has been extensively popularized in many books and media (43, 44). However, accumulating evidence indicates that the proposed enzymatic competition between ω-6, specially LA, and ω-3 PUFAs does not translate into a functionally relevant antagonism under typical dietary conditions in humans, challenging the notion that dietary LA is inherently pro-inflammatory or pro-atherogenic (45–47). Moreover, the conversion of dietary LA to harmful derivatives may be influenced by the prevailing oxidative and inflammatory state (48, 49).

Although the *ω*-6/ω-3 ratio has been questioned due to its limited ability to capture metabolic variability among individual fatty acids, it remains a topic of debate in the literature. Historically, population-level interventions such as the North Karelia Project demonstrated substantial reductions in serum cholesterol and cardiovascular mortality following comprehensive lifestyle changes, notably the replacement of saturated fat–rich foods with vegetable oils characterized by a more favorable *ω*-3/ω-6 profile and increased fish consumption (50, 51). However, these benefits cannot be attributed solely to changes in *ω*-6/ω-3 ratio, as they occurred alongside major reductions in saturated fat consumption and other lifestyle modifications.

Within this population-based and epidemiological context, contemporary research has examined dietary and circulating ω-6/ω-3 ratios as integrative markers of PUFA balance and cardiometabolic risk. Several reviews and observational studies report associations between lower ratios and more favorable inflammatory and cardiometabolic profiles, supporting its use as a descriptive indicator of lipid quality (52, 53). In parallel, both *ω*-6 and ω-3 PUFAs are widely recognized as essential for human health; however, modern Western dietary patterns are characterized by a marked imbalance favoring *ω*-6 intake, underscoring the need to raise awareness among both the general population and health professionals to promote a healthier balance (54). Accordingly, the *ω*-6/ω-3 ratio remains widely used in nutritional and cardiovascular research, although its optimal value and contextual applicability remain controversial (54, 55). Large-scale cohort studies suggest that the ω-6/ω-3 ratio functions primarily as a descriptive biomarker rather than a mechanistic determinant of cardiovascular risk, with both omega-6, particularly LA, and omega-3 fatty acids individually showing inverse associations with mortality (56, 57). This distinction underscores the need to interpret the ratio within a broader biological context and to reconsider the view of dietary LA as inherently harmful, as evidence discussed later does not support an association with adverse cardiometabolic health outcomes (58, 59).

Consequently, recent literature increasingly favors analyses focusing on individual fatty acids. In line with this perspective, clinical and lipidomic studies indicate that cardiometabolic effects depend on specific fatty acid species, their lipid compartmentalization, and their conversion to bioactive mediators, rather than on global PUFA ratios (60). Thus, while the *ω*-6/ω-3 ratio may retain descriptive value, it should be interpreted cautiously and complemented by species-level and mechanistic analyses.

## 3 Dietary linoleic acid: intake and historical recommendations

Dietary linoleic acid (LA) is the most abundant PUFA in modern diets, providing about 6% of daily energy in the average US diet (14 g in a diet of 2000 kcal/day), roughly 10 times higher than ALA and 100 times higher than long-chain PUFAs such as AA, EPA, and DHA (61). However, recommendations for *ω*-6 PUFA intake remain debated, largely due to concerns that high LA consumption may favor AA synthesis and its inflammatory derivatives at the expense of EPA and DHA (62). However, intakes above 2% of calories do not significantly increase AA levels, as its production saturates at low LA intake (24, 25). Consequently, conversion of LA to AA and tissue enrichment may be minimal, and higher LA intake in humans does not increase inflammatory markers (45, 63, 64) and may even confer cardiometabolic benefits, as detailed below. In fact, the nutrition subcommittee of the American Heart Association published in 2009 a science advisory recommending not reducing *ω*-6 PUFA intake (65).

Initial estimates of LA requirements in humans date back to the 1950s, when low-fat infant diets caused reversible skin alterations that were corrected by supplementing 2% LA as trilinolein (66). Subsequent studies suggested a minimum requirement of 1% of caloric intake and an optimal intake of around 4% (67). In the same decade, the Seven Countries Study led by Ancel Keys promoted increased consumption of LA-rich vegetable oils to lower cholesterol and cardiovascular risk (68), contributing to a dramatic rise in the consumption of these oils in the United States (69). Between 1909 and 1999, per-capita vegetable oil intake increased from 0.7 to 14.7 kg/person/year (over a 20-fold rise), driven mainly by soybean oil, which rose from 0.01 to 11.6 kg/person/year. As a result, the percentage of energy derived LA increased from 2.2–2.8 to 7.2%, representing a 158–223% rise based on the model used (69).

Over subsequent decades, international organizations established recommendations aimed at reducing cardiovascular disease risk. The AHA currently advises 5–10% of daily energy from *ω*-6 PUFAs (65), NAM and DGA 2020–2025 set adequate intakes of 12 g/day for women and 17 g/day for men aged 19–50 (5–6% of energy) (70), and FAO/WHO recommends 2.5–9% of energy consumption derived from LA (71). While evidence supports these guidelines, the impact of LA intake may vary depending on its dietary source and the physiological context. Importantly, the consumption of vegetable oils may exert biological effects beyond their fatty acid composition, as these oils contain minor bioactive compounds with potential anti-inflammatory and cardioprotective properties. For example, the health effects attributed to olive oil consumption have been shown to be influenced not only by its fatty acid profile but also by phenolic compounds such as oleocanthal (72). In addition, the cardiometabolic impact of increasing PUFA intake appears to be context-dependent, such that replacing saturated fat–rich foods with PUFA-containing vegetable oils may be beneficial when baseline saturated fat intake is high, whereas the effects may differ in dietary patterns already low in saturated fats (73).

## 4 Associations between linoleic acid and cardiometabolic outcomes: evidence in humans

Observational cohorts, biomarker-based studies, clinical trials, and genetic analyses have evaluated the association between LA intake or circulating levels and various cardiometabolic outcomes.

Already in 2007, Harris et al. showed that higher blood or tissue LA content was associated with a lower risk of non-fatal coronary events events in a meta-analysis of case–control or prospective cohort datasets (58). A comprehensive multivariable-adjusted meta-analysis based on individual LA measurements in 30 prospective observational studies from 13 countries later confirmed that higher circulating concentrations of LA were significantly associated with a reduced risk of total cardiovascular disease, stroke and cardiovascular mortality (74). Similarly, the Cardiovascular Health Study—a prospective cohort of older adults—showed that higher circulating levels of LA, but not other omega-6 fatty acids, were inversely associated with total mortality, mostly explained by lower cardiovascular mortality due to coronary heart disease (75). Taken together, these findings suggest a beneficial role for LA in CVD prevention.

Regarding dietary intake, the evidence supporting its benefits has been less consistent. One of the few intervention trials specifically targeting LA intake was the Sydney Diet Heart Study, conducted in the late 1960s and published in 1978 (76), which replaced saturated fats with safflower oil in men with coronary disease. A reanalysis of the trial decades later suggested higher mortality in the intervention group (35), but these findings have been considered unreliable due to major methodological limitations, including outdated clinical context. In addition, a systematic review of 19 randomized controlled trials found that increasing omega-6 fat intake reduced total cholesterol but had no clear effect on overall cardiovascular outcomes, except for a possible modest reduction in myocardial infarction risk (77). In contrast, Wang et al. reported a strong inverse association between LA intake and both total and cardiovascular mortality in two large US cohorts with repeated dietary assessments and long-term follow-up (59). More recently, meta-analysis of 31 prospective cohorts, showed that both higher dietary intake and circulating levels of LA were also significantly associated with a lower risk of developing type 2 diabetes (78).

In terms of underlying metabolic conditions leading to diabetes and CVD, some cross-sectional studies have reported that higher dietary linoleic acid (LA) intake is associated with greater prevalence of MetS in obese populations (79). However, a Mendelian randomization analysis found that genetically determined high circulating LA levels were inversely associated with the risk of type 2 diabetes, fasting glucose, and HbA1c, supporting a potential protective role of LA in glucose metabolism (80). Additionally, transcriptomic analysis of adipose tissue from individuals with and without MetS revealed that the LA metabolism pathway is significantly downregulated in MetS. Key genes involved in this pathway—JMJD7-PLA2G4B, PLA2G1B, PLA2G2D, CYP2C8, and CYP2J2—were all downregulated in the MetS group (81). These genes regulate the release and biotransformation of LA into bioactive metabolites; thus their reduced expression suggests impaired LA metabolism and low levels on LA-derived biomarkers, potentially contributing to metabolic dysfunction. In fact, an interventional study in postmenopausal women with MetS demonstrated that consumption of LA-rich oil increased circulating oxylipins and increased adiponectin levels (82)—implying beneficial metabolic effects, even though the clinical relevance of these findings remains to be fully established.

This diversity in findings in humans likely stems from differences in study populations, dietary contexts, compensatory nutrient intake, and metabolic variability. Indeed, recent studies suggest that the metabolic response to LA intake—including potential benefits and harms—may depend on polymorphisms in the FADS1 gene (83, 84).

In summary, human evidence generally supports a favorable role for LA in cardiometabolic health, but findings vary across populations and outcomes. The observed heterogeneity underscores the need for more well-controlled, mechanistically informed studies that account for genetic background, diet composition, and underlying metabolic status of participants.

## 5 Proposed mechanisms for linoleic acid-dependent effects on cardiometabolic health

The molecular effect of LA and other *ω*-6 PUFA on cardiometabolic health is not entirely clear (Table 1). Conflicting findings suggest that its impact may vary across different metabolic disorders, likely due to dietary complexity and individual variability.

**Table 1:** Biological processes and molecular pathways linking linoleic acid to cardiometabolic health.

Functional category	Molecular pathway	Physiological consequence	Reference
Glucose metabolism	PPARγ	LA → PPARγ: Increased insulin sensitivity.	(80, 88)
		13-HODE → PPARγ: Regulates genes involved in glucose metabolism.	(31)
		PPARγ → GLUT4: Increased glucose uptake in muscle and adipose tissue.	(95, 96)
Lipid metabolism	PPARγ activation: Coordinated regulation of cholesterol metabolism	LXRα (hepatic): Increased bile acid synthesis and hepatic clearance of cholesterol, as well as decreasing LDL-cholesterol.	(89, 90)
		LDLR: Increased hepatic LDL uptake and decreased circulating cholesterol.	(91)
		LXR → ABCA1: Increased cholesterol efflux and HDL formation.	(88)
		SR-B1: Increased HDL cholesterol uptake and altered HDL particle size in rat hepatocytes.	(101)
	Fatty acid oxidation (BAT, muscle)	12,13-DiHOME enhances fatty acid uptake and oxidation during cold or exercise → Lower triglyceride levels and improved metabolic flexibility and vascular repair.	(33, 34)
Inflammation	PPARγ	PPARγ activation inhibits NF-κB (↑ IκBα, transrepression) and decreased pro-inflammatory cytokines (IL-6, TNF-α, MCP-1).	(30)
		13-HODE → PPARγ: Reduction of endothelial adhesion associated with anti-atherogenic effects.	(100, 101)
		13-HODE → PPARγ suppresses NF-κB activation and modulates IL-2 production. Reduced inflammation and immune homeostasis.	(32)
Oxidative stress	Cardiolipins	LA-rich cardiolipins → highly prone to oxidation. This leads to mitochondrial dysfunction, apoptosis and inflammation.	(105)
	LDL particles	LA is predominant fatty acid → prone to peroxidation. Formation of oxidized LDL (OXLAMs) and ↑ atherogenesis.	(106, 107)
Microbiota-host metabolic interaction	Gut microbiota (mice)	High-LA diets can induce dysbiosis, increase susceptibility to colitis and disrupt intestinal endocannabinoid signaling.	(109)
		*Lactobacillus* strains metabolize LA into hydroxy fatty acids such as HYA. Exert protective effects against diet-induced obesity and glucose intolerance.	(110)
	Gut microbiota (humans)	Plasma LA levels are inversely associated with Gestational Diabetes risk, mediated by microbial taxa (e.g., *Bilophila wadsworthia*). → Microbiota-dependent metabolic protection through improved glucose regulation.	(111)

CVD and related metabolic conditions—such as obesity, MetS, and T2D—share common pathogenic mechanisms including dyslipidemia, insulin resistance, inflammation, and endothelial dysfunction. LA has been suggested to reduce the risk of CVDs by lowering total blood cholesterol compared to Western diets or diets high in SFAs (74, 77, 85–87). However, its effects on lipoprotein subfractions vary depending on the comparator diet and study design. For example, LA-enriched diets tend to decrease LDL cholesterol and increase HDL cholesterol relative to diets high in SFAs or trans fatty acids (TFAs), while results are inconsistent when compared with MUFA- or omega-3 PUFA-rich diets (85). One randomized, double-blind crossover study comparing omega-6 (mainly LA) and omega-3 supplementation showed that omega-6 reduced total cholesterol, LDL-C, and ApoB, the main atherogenic apolipoprotein, without affecting HDL-C or triglycerides (86). Interestingly, omega-6 supplementation increased small HDL particles at the expense of larger ones, whereas omega-3 supplementation lowered triglycerides and increased HDL-C without altering LDL-C or total cholesterol (86).

The lipid-modifying effects of LA likely involve regulation of key transcription factors controlling lipid metabolism, such as peroxisome proliferator-activated receptors (PPARs) and liver X receptors (LXRs) (88, 89). Although few studies have isolated the effects of LA *per se*, evidence suggests that LA’s lipid-lowering action is mediated in part by LXR. For instance, diets supplemented with soy oil (rich in LA) increased hepatic LXRα mRNA and protein levels in rats (89). LXR activation promotes cholesterol homeostasis by inducing cholesterol 7-hydroxylase (CYP7), the enzyme catalyzing the conversion of cholesterol into bile acids, facilitating LDL-C clearance (90). In fact, LA intake has also been shown to increase LDL receptor (LDLR) expression in young pigs, enhancing hepatic LDL uptake and lowering circulating LDL-C (91). Additionally, LXR activation by LA and its oxidized metabolites may induce ATP-binding cassette transporter A1 (ABCA1) expression, a critical mediator of cholesterol efflux and HDL formation, which may also contribute to atheroprotection (92, 93).

These pathways provide a plausible basis for LA’s lipid-lowering effects, yet they do not fully explain its potential benefits on glucose metabolism. *In vitro* evidence indicates that LA can bind and activate PPARγ, a nuclear receptor central to adipogenesis, insulin sensitivity, and glucose homeostasis (94). PPARγ activation improves whole-body insulin sensitivity by increasing GLUT4 gene expression and translocation to the membrane in adipose tissue and skeletal muscle, enhancing glucose uptake (95, 96). This potential LA-PPARγ-glucose metabolism connection may partially explain the inverse association between circulating LA and T2DM risk reported in prospective cohort studies (78). Notably, the LA-derived oxidized metabolites 9-HODE and 13-HODE have also been identified as natural ligands of PPARγ, suggesting that their effects on lipid and glucose metabolism, adiponectin secretion, and inflammatory signaling may be mediated through PPARγ activation (97). Furthermore, PPARγ inhibits the pro-inflammatory transcription factor NF-κB, reducing cytokine expression such as IL-6, TNF-*α*, and MCP-1 (98), which may also contribute to the cardioprotective effects attributed to LA. In addition, PPARγ activation stimulates the expression of LDLR, LXR, and SR-B1 (99–101), a key receptor for HDL cholesterol uptake, possibly explaining the changes in HDL particle size observed with LA intake (86).

Interestingly, the relationship between PPARγ and NF-κB illustrates a critical regulatory node: PPARγ activation suppresses NF-κB-mediated inflammation through upregulation of IκBα and transrepression mechanisms (102). However, under conditions of oxidative stress, LA-derived oxidized metabolites can activate NF-κB, promoting chronic low-grade inflammation and metabolic dysfunction (103, 104). Therefore, this biphasic effect underscores the dual role of LA in inflammation and highlights the importance of biological context when understanding the pathophysiological effects of this omega-6 PUFA.

At the mitochondrial level, LA is a major fatty acid constituent of cardiolipins, which are essential phospholipids for mitochondrial membrane integrity and function. Oxidation of LA-rich cardiolipins impairs mitochondrial respiration and triggers apoptotic and inflammatory cascades, exacerbating metabolic disturbances (105). Additionally, LA is the predominant fatty acid in native LDL particles and is highly prone to peroxidation. *In vitro*, oxidized LA metabolites accumulate in oxidized LDL (106, 107), a modified particle known to be highly atherogenic and implicated in atherosclerosis development (108). Although causal links between dietary LA, LA content in LDL particles, and atherosclerosis remain unproven, these findings highlight the importance of oxidative stress, inflammation, and aging in modulating LA’s effects on cardiometabolic health.

Therefore, LA may contribute to cardiometabolic health through multiple interconnected mechanisms involving nuclear receptor signaling (PPARγ, LXR), modulation of lipid metabolism (LDLR, ABCA1, SR-B1), glucose uptake (GLUT4), and anti-inflammatory effects (NF-κB inhibition). However, these benefits may be compromised under pro-oxidative conditions, emphasizing the context-dependent nature of LA’s biological actions.

Lastly, emerging evidence suggests that dietary LA can influence gut microbiota and, through this pathway, modulate host metabolic and inflammatory outcomes. In murine models, high-LA diets have been shown to induce dysbiosis, increase susceptibility to colitis, and disrupt intestinal endocannabinoid signaling (109), whereas certain *Lactobacillus* strains metabolize LA into hydroxy fatty acids such as 10-hydroxy-cis-12-octadecenoic acid (HYA) that exert protective effects against diet-induced obesity and glucose intolerance (110). In humans, the evidence is still scarce. A case–cohort study has reported that higher plasma LA levels during pregnancy were inversely associated with Gestational Diabetes Mellitus risk, and that this protective effect was both mediated and modified by gut microbial taxa, including *Bilophila wadsworthia* (111). While these findings highlight the potential for LA–microbiota interactions to shape outcomes ranging from intestinal inflammation to metabolic health, further controlled dietary interventions are required to establish causality in humans.

## 6 Discussion

In summary, linoleic acid should not be considered inherently harmful. Rather, its impact on cardiometabolic health depends on dietary context, metabolic status, and downstream metabolism. More broadly, dietary influences extend beyond individual fatty acids and reflect the complexity of whole foods and dietary patterns.

Despite the global burden of cardiometabolic diseases, the specific role of dietary linoleic acid in their prevention remains insufficiently defined. While LA is the predominant dietary *ω*-6 PUFA, misconceptions about its impact have arisen from incomplete understanding of its metabolism and downstream effects. In particular, the widespread belief that high ω-6 PUFA intake is inherently deleterious has led to calls for restricting LA consumption, despite the lack of robust scientific evidence to support such recommendation. Contrary to this view, LA does not appear to impair ω-3 PUFA status, and multiple studies suggest cardiometabolic benefits of higher LA consumption. Within this context, the commonly cited omega-6/omega-3 ratio has limited mechanistic relevance and may oversimplify PUFA metabolism, although it can retain descriptive utility in specific dietary or population-based contexts. Most human evidence supports a protective association between LA intake and cardiovascular and metabolic outcomes, yet the potential adverse effects of its oxidized derivatives warrant further investigation. This duality emphasizes the importance of context: while LA shows promising effects on lipid profiles, inflammation, glucose metabolism, and cardiovascular health—particularly relevant for patients with MetS in whom cardiovascular and T2D risks converge—heterogeneity across experimental designs, populations, and mechanistic insights leaves critical gaps. In this context, ongoing intervention studies, including a recently initiated clinical trial (ClinicalTrials.gov identifier: NCT07287514), are designed to directly examine the effects of dietary linoleic acid intake on cardiometabolic biomarkers and may help clarify its role within specific metabolic contexts. Continued integration of mechanistic, observational, and interventional research will be essential to refine dietary recommendations and to fully elucidate the role of LA in cardiometabolic disease prevention.

## Glossary

AA: Arachidonic acid
ABCA1: ATP-binding cassette transporter A1
AHA: American Heart Association
ALA: *α*-Linolenic acid
ApoB: Apolipoprotein B
CVD: Cardiovascular disease
CYP: Cytochrome P450
DHA: Docosahexaenoic acid
DiHOME: Dihydroxyoctadecenoic acid
EPA: Eicosapentaenoic acid
FADS: Fatty acid desaturase
HDL-C: High-density lipoprotein cholesterol
HEI: Healthy Eating Index
HODE: Hydroxyoctadecadienoic acid
HYA: 10-hydroxy-cis-12-octadecenoic acid
LA: Linoleic acid
LDL-C: Low-density lipoprotein cholesterol
LDLR: Low-density lipoprotein receptor
LXR: Liver X receptor
MetS: Metabolic syndrome
MUFA: Monounsaturated fatty acid
NF-κB: Nuclear factor kappa-light-chain-enhancer of activated B cells
NCD: Non-communicable disease
OXLAM: Oxidized linoleic acid metabolite
oxylipins: Oxidized lipid mediators derived from PUFA
PPARγ: Peroxisome proliferator-activated receptor gamma
PUFA: Polyunsaturated fatty acid
SFA: Saturated fatty acid
sEH: soluble Epoxide Hydrolase
SR-B1: Scavenger receptor class B type 1
TG: Triglycerides
T2D: Type 2 diabetes mellitus

## References

[1] WHO. Noncommunicable diseases: key facts: World Health Organization; (2025). Available online at: https://www.who.int/news-room/fact-sheets/detail/noncommunicable-diseases (Accessed October 15, 2025).

[2] Alberti KGMM Eckel RH Grundy SM Zimmet PZ Cleeman JI Donato KA . Harmonizing the metabolic syndrome: a joint interim statement of the international diabetes federation task force on epidemiology and prevention; National Heart, Lung, and Blood Institute; American Heart Association; world heart federation; international atherosclerosis society; and International Association for the Study of obesity. Circulation. (2009) 120:1640–5. doi: 10.1161/CIRCULATIONAHA.109.192644, 19805654

[3] Minsal. ENCUESTA NACIONAL DE SALUD 2016–2017 Primeros resultados. Chile: Gobierno de Chile (2017).

[4] Noubiap JJ Nansseu JR Lontchi-Yimagou E Nkeck JR Nyaga UF Ngouo AT . Global, regional, and country estimates of metabolic syndrome burden in children and adolescents in 2020: a systematic review and modelling analysis. Lancet Child & Adolescent Health. (2022) 6:158–70. doi: 10.1016/S2352-4642(21)00374-6, 35051409

[5] Després JP. Is visceral obesity the cause of the metabolic syndrome? Ann Med. (2006) 38:52–63. doi: 10.1080/07853890500383895, 16448989

[6] Hagberg CE Spalding KL. White adipocyte dysfunction and obesity-associated pathologies in humans. Nat Rev Mol Cell Biol. (2024) 25:270–89. doi: 10.1038/s41580-023-00680-1, 38086922

[7] Zatterale F Longo M Naderi J Raciti GA Desiderio A Miele C . Chronic adipose tissue inflammation linking obesity to insulin resistance and type 2 diabetes. Front Physiol. (2019) 10:1607. doi: 10.3389/fphys.2019.01607 32063863 PMC7000657

[8] Murray CJ Atkinson C Bhalla K Birbeck G Burstein R Chou D . The state of US health, 1990-2010: burden of diseases, injuries, and risk factors. JAMA. (2013) 310:591–608. doi: 10.1001/jama.2013.13805, 23842577 PMC5436627

[9] Willett WC Stampfer MJ. Current evidence on healthy eating. Annu Rev Public Health. (2013) 34:77–95. doi: 10.1146/annurev-publhealth-031811-124646, 23297654

[10] Jacques PF Tucker KL. Are dietary patterns useful for understanding the role of diet in chronic disease? Am J Clin Nutr. (2001) 73:1–2. doi: 10.1093/ajcn/73.1.1, 11124739

[11] Bhupathiraju SN Tucker KL. Coronary heart disease prevention: nutrients, foods, and dietary patterns. Clin Chim Acta. (2011) 412:1493–514. doi: 10.1016/j.cca.2011.04.038, 21575619 PMC5945285

[12] Katan MB Grundy SM Willett WC. Should a low-fat, high-carbohydrate diet be recommended for everyone? Beyond low-fat diets. N Engl J Med. (1997) 337:562–7. 9262504

[13] Liu AG Ford NA Hu FB Zelman KM Mozaffarian D Kris-Etherton PM. A healthy approach to dietary fats: understanding the science and taking action to reduce consumer confusion. Nutr J. (2017) 16:53. doi: 10.1186/s12937-017-0271-4, 28854932 PMC5577766

[14] Aronne LBK Campbell A Coleman L. Best diets overall 2023 health U.S. Dent News. (2023) Available online at: https://health.usnews.com/best-diet/best-diets-overall (Accessed October 15, 2025).

[15] Estruch R Martínez-González MA Corella D Salas-Salvadó J Fitó M Chiva-Blanch G . Effect of a high-fat Mediterranean diet on bodyweight and waist circumference: a prespecified secondary outcomes analysis of the PREDIMED randomised controlled trial. Lancet Diabetes Endocrinol. (2019) 7:e6–e17. doi: 10.1016/S2213-8587(19)30074-9, 31003626

[16] Riolo R De Rosa R Simonetta I Tuttolomondo A. Olive oil in the Mediterranean diet and its biochemical and molecular effects on cardiovascular health through an analysis of genetics and epigenetics. Int J Mol Sci. (2022) 23:002. doi: 10.3390/ijms232416002, 36555645 PMC9782563

[17] Wu H Xu L Ballantyne CM. Dietary and pharmacological fatty acids and cardiovascular health. J Clin Endocrinol Metab. (2020) 105:1030–45. doi: 10.1210/clinem/dgz174, 31678992 PMC7174038

[18] Kaur N Chugh V Gupta AK. Essential fatty acids as functional components of foods- a review. J Food Sci Technol. (2014) 51:2289–303. doi: 10.1007/s13197-012-0677-0, 25328170 PMC4190204

[19] Calder PC. Functional roles of fatty acids and their effects on human health. JPEN J Parenter Enteral Nutr. (2015) 39:18s–32s. doi: 10.1177/0148607115595980, 26177664

[20] Sun L Zong G Li H Lin X. Fatty acids and cardiometabolic health: a review of studies in Chinese populations. Eur J Clin Nutr. (2021) 75:253–66. doi: 10.1038/s41430-020-00709-0, 32801302

[21] Whelan J Fritsche K. Linoleic acid. Adv Nutr. (2013) 4:311–2. doi: 10.3945/an.113.003772, 23674797 PMC3650500

[22] Whelan J. The health implications of changing linoleic acid intakes. Prostaglandins Leukot Essent Fat Acids. (2008) 79:165–7. doi: 10.1016/j.plefa.2008.09.013, 18990554

[23] Dyall SC Balas L Bazan NG Brenna JT Chiang N da Costa Souza F . Polyunsaturated fatty acids and fatty acid-derived lipid mediators: recent advances in the understanding of their biosynthesis, structures, and functions. Prog Lipid Res. (2022) 86:101165. doi: 10.1016/j.plipres.2022.101165, 35508275 PMC9346631

[24] James MJ Gibson RA D'Angelo M Neumann MA Cleland LG. Simple relationships exist between dietary linoleate and the n-6 fatty acids of human neutrophils and plasma. Am J Clin Nutr. (1993) 58:497–500. 8379505 10.1093/ajcn/58.4.497

[25] Mantzioris E James MJ Gibson RA Cleland LG. Differences exist in the relationships between dietary linoleic and alpha-linolenic acids and their respective long-chain metabolites. Am J Clin Nutr. (1995) 61:320–4. 7840069 10.1093/ajcn/61.2.320

[26] Santos HO Price JC Bueno AA. Beyond fish oil supplementation: the effects of alternative plant sources of Omega-3 polyunsaturated fatty acids upon lipid indexes and Cardiometabolic biomarkers-an overview. Nutrients. (2020) 12:159. doi: 10.3390/nu12103159, 33081119 PMC7602731

[27] Mustonen AM Nieminen P. Dihomo-γ-linolenic acid (20:3n-6)-metabolism, derivatives, and potential significance in chronic inflammation. Int J Mol Sci. (2023) 24:116. doi: 10.3390/ijms24032116, 36768438 PMC9916522

[28] Ağagündüz D Yeşildemir Ö Koçyiğit E Koçak T Özen Ünaldı B Ayakdaş G . Oxylipins derived from PUFAs in Cardiometabolic diseases: mechanism of actions and possible nutritional interactions. Nutrients. (2024) 16:812. doi: 10.3390/nu16223812, 39599599 PMC11597274

[29] Gabbs M Leng S Devassy JG Monirujjaman M Aukema HM. Advances in our understanding of Oxylipins derived from dietary PUFAs. Adv Nutr. (2015) 6:513–40. doi: 10.3945/an.114.007732, 26374175 PMC4561827

[30] Vangaveti VN Jansen H Kennedy RL Malabu UH. Hydroxyoctadecadienoic acids: oxidised derivatives of linoleic acid and their role in inflammation associated with metabolic syndrome and cancer. Eur J Pharmacol. (2016) 785:70–6. doi: 10.1016/j.ejphar.2015.03.096, 25987423

[31] Clarke SD Thuillier P Baillie RA Sha X. Peroxisome proliferator-activated receptors: a family of lipid-activated transcription factors. Am J Clin Nutr. (1999) 70:566–71. 10500027 10.1093/ajcn/70.4.566

[32] Kühn H O'Donnell VB. Inflammation and immune regulation by 12/15-lipoxygenases. Prog Lipid Res. (2006) 45:334–56. doi: 10.1016/j.plipres.2006.02.003, 16678271

[33] Lynes MD Leiria LO Lundh M Bartelt A Shamsi F Huang TL . The cold-induced lipokine 12,13-diHOME promotes fatty acid transport into brown adipose tissue. Nat Med. (2017) 23:631–7. doi: 10.1038/nm.4297, 28346411 PMC5699924

[34] Hildreth K Kodani SD Hammock BD Zhao L. Cytochrome P450-derived linoleic acid metabolites EpOMEs and DiHOMEs: a review of recent studies. J Nutr Biochem. (2020) 86:108484. doi: 10.1016/j.jnutbio.2020.108484, 32827665 PMC7606796

[35] Ramsden CE Zamora D Leelarthaepin B Majchrzak-Hong SF Faurot KR Suchindran CM . Use of dietary linoleic acid for secondary prevention of coronary heart disease and death: evaluation of recovered data from the Sydney diet heart study and updated meta-analysis. BMJ. (2013) 346:e8707. doi: 10.1136/bmj.e8707, 23386268 PMC4688426

[36] Ochin CC Wilson T Garelnabi M. Dietary oxidized linoleic acids modulate fatty acids in mice. J Lipid Atheroscler. (2022) 11:197–210. doi: 10.12997/jla.2022.11.2.197, 35656146 PMC9133782

[37] Lee JM Lee H Kang S Park WJ. Fatty acid desaturases, polyunsaturated fatty acid regulation, and biotechnological advances. Nutrients. (2016) 8:023. doi: 10.3390/nu8010023, 26742061 PMC4728637

[38] Simopoulos AP. Importance of the omega-6/omega-3 balance in health and disease: evolutionary aspects of diet. World Rev Nutr Diet. (2011) 102:10–21. doi: 10.1159/000327785, 21865815

[39] Simopoulos AP Leaf A Salem N Jr. Essentiality of and recommended dietary intakes for omega-6 and omega-3 fatty acids. Ann Nutr Metab. (1999) 43:127–30. 10436312 10.1159/000012777

[40] Simopoulos AP. The importance of the omega-6/omega-3 fatty acid ratio in cardiovascular disease and other chronic diseases. Exp Biol Med (Maywood). (2008) 233:674–88. doi: 10.3181/0711-MR-311, 18408140

[41] Simopoulos AP. Omega-6/omega-3 essential fatty acids: biological effects. World Rev Nutr Diet. (2009) 99:1–16. doi: 10.1159/000192755 19136835

[42] Simopoulos AP. Evolutionary aspects of the dietary omega-6:omega-3 fatty acid ratio: medical implications. World Rev Nutr Diet. (2009) 100:1–21. doi: 10.1159/000235706, 19696523

[43] Willington C. The omega ratio miracle: Feel better, look better and live longer with a BALANCED OMEGA-6 to OMEGA-3 ratio. UNABRIDGED EDITION ed. New York (NY): HarperCollins Publishers. (2024).

[44] Rogers K. Balance omega-3 and 6 intake to cut early death risk, study suggests. CNN. (2024) Available online at: https://edition.cnn.com/2024/05/14/health/omega-3-omega-6-death-risk-wellness (Accessed October 15, 2025).

[45] Fritsche KL. Too much linoleic acid promotes inflammation-doesn't it? Prostaglandins Leukot Essent Fat Acids. (2008) 79:173–5. doi: 10.1016/j.plefa.2008.09.019, 18990555

[46] Harris WS Shearer GC. Omega-6 fatty acids and cardiovascular disease: friend, not foe? Circulation. (2014) 130:1562–4. doi: 10.1161/CIRCULATIONAHA.114.012534, 25161044

[47] Harris WS. The Omega-6:Omega-3 ratio: a critical appraisal and possible successor. Prostaglandins Leukot Essent Fat Acids. (2018) 132:34–40. doi: 10.1016/j.plefa.2018.03.003, 29599053

[48] Calder PC. Polyunsaturated fatty acids and inflammatory processes: new twists in an old tale. Biochimie. (2009) 91:791–5. doi: 10.1016/j.biochi.2009.01.008, 19455748

[49] Kwon SY Massey K Watson MA Hussain T Volpe G Buckley CD . Oxidised metabolites of the omega-6 fatty acid linoleic acid activate dFOXO. Life Sci Alliance. (2020) 3:e201900356. doi: 10.26508/lsa.201900356, 31992650 PMC6988086

[50] Puska P Vartiainen E Tuomilehto J Salomaa V Nissinen A. Changes in premature deaths in Finland: successful long-term prevention of cardiovascular diseases. Bull World Health Organ. (1998) 76:419–25. 9803593 PMC2305767

[51] Puska P Jaini P. The North Karelia project: prevention of cardiovascular disease in Finland through population-based lifestyle interventions. Am J Lifestyle Med. (2020) 14:495–9. doi: 10.1177/1559827620910981, 32922234 PMC7444010

[52] Djuricic I Calder PC. Beneficial outcomes of Omega-6 and Omega-3 polyunsaturated fatty acids on human health: an update for 2021. Nutrients. (2021) 13:421. doi: 10.3390/nu13072421, 34371930 PMC8308533

[53] Gonzalez-Becerra K Barron-Cabrera E Muñoz-Valle JF Torres-Castillo N Rivera-Valdes JJ Rodriguez-Echevarria R . A Balanced dietary ratio of n-6:n-3 polyunsaturated fatty acids exerts an effect on Total fatty acid profile in RBCs and inflammatory markers in subjects with obesity. Health. (2023) 11:333. doi: 10.3390/healthcare11162333, 37628530 PMC10454033

[54] Gutierres D Pacheco R Reis CP. The role of Omega-3 and Omega-6 polyunsaturated fatty acid supplementation in human health. Foods. (2025) 14:299. doi: 10.3390/foods14193299, 41097470 PMC12524211

[55] D'Angelo S Motti ML Meccariello R. Ω-3 and ω-6 polyunsaturated fatty acids, obesity and cancer. Nutrients. (2020) 12:2751. doi: 10.3390/nu12092751 PMC7551151 32927614

[56] Huang Y Lu X Shen Y Liu Y Zeng Q Liu X . The NMR-measured omega-6/omega-3 fatty acid ratio improves cardiovascular risk prediction. Front Nutr. (2025) 12:1693151. doi: 10.3389/fnut.2025.1693151, 41235302 PMC12605120

[57] Zhang Y Sun Y Yu Q Song S Brenna JT Shen Y . Higher ratio of plasma omega-6/omega-3 fatty acids is associated with greater risk of all-cause, cancer, and cardiovascular mortality: a population-based cohort study in UK biobank. eLife. (2024) 12:12. doi: 10.7554/eLife.90132.3, 38578269 PMC10997328

[58] Harris WS Poston WC Haddock CK. Tissue n-3 and n-6 fatty acids and risk for coronary heart disease events. Atherosclerosis. (2007) 193:1–10. doi: 10.1016/j.atherosclerosis.2007.03.018, 17507020

[59] Wang DD Li Y Chiuve SE Stampfer MJ Manson JE Rimm EB . Association of specific dietary fats with total and cause-specific mortality. JAMA Intern Med. (2016) 176:1134–45. doi: 10.1001/jamainternmed.2016.2417, 27379574 PMC5123772

[60] Mahmoud AM Mirza I Metwally E Morsy MH Scichilone G Asada MC . Lipidomic profiling of human adiposomes identifies specific lipid shifts linked to obesity and cardiometabolic risk. JCI Insight. (2025) 10:872. doi: 10.1172/jci.insight.191872, 40548377 PMC12226050

[61] Jandacek RJ. Linoleic acid: a nutritional quandary. Healthcare (Basel). (2017) 5:025. doi: 10.3390/healthcare5020025, 28531128 PMC5492028

[62] DiNicolantonio JJ O'Keefe J. The importance of maintaining a low omega-6/omega-3 ratio for reducing the risk of inflammatory cytokine storms. Mo Med. (2020) 117:539–42. 33311785 PMC7721408

[63] Rett BS Whelan J. Increasing dietary linoleic acid does not increase tissue arachidonic acid content in adults consuming Western-type diets: a systematic review. Nutr Metab (Lond). (2011) 8:36. doi: 10.1186/1743-7075-8-36, 21663641 PMC3132704

[64] Johnson GH Fritsche K. Effect of dietary linoleic acid on markers of inflammation in healthy persons: a systematic review of randomized controlled trials. J Acad Nutr Diet. (2012) 112:1029–41. doi: 10.1016/j.jand.2012.03.029 22889633

[65] Harris WS Mozaffarian D Rimm E Kris-Etherton P Rudel LL Appel LJ . Omega-6 fatty acids and risk for cardiovascular disease: a science advisory from the American Heart Association nutrition Subcommittee of the Council on nutrition, physical activity, and metabolism; council on cardiovascular nursing; and council on epidemiology and prevention. Circulation. (2009) 119:902–7. doi: 10.1161/CIRCULATIONAHA.108.191627 19171857

[66] Hansen AE Haggard ME Boelsche AN Adam DJ Wiese HF. Essential fatty acids in infant nutrition. III. Clinical manifestations of linoleic acid deficiency. J Nutr. (1958) 66:565–76. 13621281 10.1093/jn/66.4.565

[67] Wiese HF Hansen AE Adam DJ. Essential fatty acids in infant nutrition. I. Linoleic acid requirement in terms of serum di-, tri- and tetraenoic acid levels. J Nutr. (1958) 66:345–60. 13611579 10.1093/jn/66.3.345

[68] Keys A. Coronary heart disease in seven countries. 1970. Nutrition. (1997) 13:250–2. 9131696 10.1016/s0899-9007(96)00410-8

[69] Blasbalg TL Hibbeln JR Ramsden CE Majchrzak SF Rawlings RR. Changes in consumption of omega-3 and omega-6 fatty acids in the United States during the 20th century. Am J Clin Nutr. (2011) 93:950–62. doi: 10.3945/ajcn.110.006643, 21367944 PMC3076650

[70] Trumbo P Schlicker S Yates AA Poos M. Dietary reference intakes for energy, carbohydrate, fiber, fat, fatty acids, cholesterol, protein and amino acids. J Am Diet Assoc. (2002) 102:1621–30. doi: 10.1016/S0002-8223(02)90346-9, 12449285

[71] Food and Agriculture Organization; World Health Organization. Fats and fatty acids in human nutrition: report of an expert consultation. Rome: FAO. (2010). 21812367

[72] Covas MI Nyyssönen K Poulsen HE Kaikkonen J Zunft HJ Kiesewetter H . The effect of polyphenols in olive oil on heart disease risk factors: a randomized trial. Ann Intern Med. (2006) 145:333–41. doi: 10.7326/0003-4819-145-5-200609050-00006, 16954359

[73] Mozaffarian D Micha R Wallace S. Effects on coronary heart disease of increasing polyunsaturated fat in place of saturated fat: a systematic review and meta-analysis of randomized controlled trials. PLoS Med. (2010) 7:e1000252. doi: 10.1371/journal.pmed.1000252, 20351774 PMC2843598

[74] Marklund M Wu JHY Imamura F Del Gobbo LC Fretts A de Goe J . Biomarkers of dietary omega-6 fatty acids and incident cardiovascular disease and mortality. Circulation. 2019;139:2422–2436, doi: 10.1161/CIRCULATIONAHA.118.038908 30971107 PMC6582360

[75] Wu JH Lemaitre RN King IB Song X Psaty BM Siscovick DS . Circulating omega-6 polyunsaturated fatty acids and total and cause-specific mortality: the cardiovascular health study. Circulation. (2014) 130:1245–53. doi: 10.1161/CIRCULATIONAHA.114.011590, 25124495 PMC4189990

[76] Woodhill JM Palmer AJ Leelarthaepin B McGilchrist C Blacket RB. Low fat, low cholesterol diet in secondary prevention of coronary heart disease. Adv Exp Med Biol. (1978) 109:317–30. 727035 10.1007/978-1-4684-0967-3_18

[77] Hooper L Al-Khudairy L Abdelhamid AS Rees K Brainard JS Brown TJ . Omega-6 fats for the primary and secondary prevention of cardiovascular disease. Cochrane Database Syst Rev. (2018) 2018:094. doi: 10.1002/14651858.CD011094.pub4 PMC6513455 30019765

[78] Mousavi SM Jalilpiran Y Karimi E Aune D Larijani B Mozaffarian D . Dietary intake of linoleic acid, its concentrations, and the risk of type 2 diabetes: a systematic review and dose-response Meta-analysis of prospective cohort studies. Diabetes Care. (2021) 44:2173–81. doi: 10.2337/dc21-0438, 34417277

[79] Maximino P Horta PM dos Santos LC de Oliveira CL Fisberg M. Fatty acid intake and metabolic syndrome among overweight and obese women. Rev Bras Epidemiol. (2015) 18:930–42. doi: 10.1590/1980-5497201500040020, 26982306

[80] Liang H Mu HB Zhang FH Li WQ Li GC Li WD . Causal relationship between linoleic acid and type 2 diabetes and glycemic traits: a bidirectional Mendelian randomization study. Front Endocrinol (Lausanne). (2023) 14:1277153. doi: 10.3389/fendo.2023.1277153, 38075067 PMC10703485

[81] Wen Y Shang Y Wang Q. Exploration of the mechanism of linoleic acid metabolism dysregulation in metabolic syndrome. Genet Res (Camb). (2022) 2022:1–7. doi: 10.1155/2022/6793346, 36518097 PMC9722286

[82] Cole RM Puchala S Ke JY Abdel-Rasoul M Harlow K O'Donnell B . Linoleic acid-rich oil supplementation increases total and high-molecular-weight adiponectin and alters plasma oxylipins in postmenopausal women with metabolic syndrome. Curr Dev Nutr. (2020) 4:nzaa136. doi: 10.1093/cdn/nzaa136, 32923921 PMC7475005

[83] Lankinen MA Fauland A Shimizu BI Ågren J Wheelock CE Laakso M . Inflammatory response to dietary linoleic acid depends on FADS1 genotype. Am J Clin Nutr. (2019) 109:165–75. doi: 10.1093/ajcn/nqy287, 30624587

[84] Vaittinen M Lankinen MA Käkelä P Ågren J Wheelock CE Laakso M . The FADS1 genotypes modify the effect of linoleic acid-enriched diet on adipose tissue inflammation via pro-inflammatory eicosanoid metabolism. Eur J Nutr. (2022) 61:3707–18. doi: 10.1007/s00394-022-02922-y, 35701670 PMC9464166

[85] Froyen E Burns-Whitmore B. The effects of linoleic acid consumption on lipid risk markers for cardiovascular disease in healthy individuals: a review of human intervention trials. Nutrients. (2020) 12:329. doi: 10.3390/nu12082329, 32759714 PMC7469037

[86] Grytten E Laupsa-Borge J Bohov P Bjørndal B Strand E Skorve J . Changes in lipoprotein particle subclasses, standard lipids, and apolipoproteins after supplementation with n-3 or n-6 PUFAs in abdominal obesity: a randomized double-blind crossover study. Clin Nutr. (2021) 40:2556–75. doi: 10.1016/j.clnu.2021.03.040, 33933722

[87] Yuan X Nagamine R Tanaka Y Tsai WT Jiang Z Takeyama A . The effects of dietary linoleic acid on reducing serum cholesterol and atherosclerosis development are nullified by a high-cholesterol diet in male and female apoE-deficient mice. Br J Nutr. (2023) 129:737–44. doi: 10.1017/S0007114522001325, 35570622

[88] Bordoni A Di Nunzio M Danesi F Biagi PL. Polyunsaturated fatty acids: from diet to binding to ppars and other nuclear receptors. Genes Nutr. (2006) 1:95–106. doi: 10.1007/BF02829951, 18850203 PMC3454679

[89] Tobin KA Steineger HH Alberti S Spydevold O Auwerx J Gustafsson JA . Cross-talk between fatty acid and cholesterol metabolism mediated by liver X receptor-alpha. Mol Endocrinol. (2000) 14:741–52. 10809236 10.1210/mend.14.5.0459

[90] Hebanowska A. Mechanisms of bile acid biosynthesis regulation--autoregulation by bile acids. Postepy Biochem. (2011) 57:314–23. 22235657

[91] Mustad VA Ellsworth JL Cooper AD Kris-Etherton PM Etherton TD. Dietary linoleic acid increases and palmitic acid decreases hepatic LDL receptor protein and mRNA abundance in young pigs. J Lipid Res. (1996) 37:2310–23. 8978483

[92] Ito A Hong C Rong X Zhu X Tarling EJ Hedde PN . LXRs link metabolism to inflammation through Abca1-dependent regulation of membrane composition and TLR signaling. eLife. (2015) 4:e08009. doi: 10.7554/eLife.08009, 26173179 PMC4517437

[93] Itoh T Fairall L Amin K Inaba Y Szanto A Balint BL . Structural basis for the activation of PPARgamma by oxidized fatty acids. Nat Struct Mol Biol. (2008) 15:924–31. doi: 10.1038/nsmb.1474, 19172745 PMC2939985

[94] Evans N Conley JM Cardon M Hartig P Medlock-Kakaley E Gray LE Jr. In vitro activity of a panel of per- and polyfluoroalkyl substances (PFAS), fatty acids, and pharmaceuticals in peroxisome proliferator-activated receptor (PPAR) alpha, PPAR gamma, and estrogen receptor assays. Toxicol Appl Pharmacol. (2022) 449:116136. doi: 10.1016/j.taap.2022.116136, 35752307 PMC9341220

[95] Tyagi S Gupta P Saini AS Kaushal C Sharma S. The peroxisome proliferator-activated receptor: a family of nuclear receptors role in various diseases. J Adv Pharm Technol Res. (2011) 2:236–40. doi: 10.4103/2231-4040.90879, 22247890 PMC3255347

[96] Bhatia V Viswanathan P. Insulin resistance and PPAR insulin sensitizers. Curr Opin Investig Drugs. (2006) 7:891–7. 17086933

[97] Belury MA. Linoleic acid, an omega-6 fatty acid that reduces risk for cardiometabolic diseases: premise, promise and practical implications. Curr Opin Clin Nutr Metab Care. (2023) 26:288–92. doi: 10.1097/MCO.0000000000000919, 37017716

[98] Yang XF Shang DJ. The role of peroxisome proliferator-activated receptor γ in lipid metabolism and inflammation in atherosclerosis. Cell Biol Int. (2023) 47:1469–87. doi: 10.1002/cbin.12065, 37369936

[99] Duan Y Chen Y Hu W Li X Yang X Zhou X . Peroxisome proliferator-activated receptor γ activation by ligands and dephosphorylation induces proprotein convertase subtilisin kexin type 9 and low density lipoprotein receptor expression. J Biol Chem. (2012) 287:23667–77. doi: 10.1074/jbc.M112.350181, 22593575 PMC3390641

[100] Chawla A Boisvert WA Lee CH Laffitte BA Barak Y Joseph SB . A PPAR gamma-LXR-ABCA1 pathway in macrophages is involved in cholesterol efflux and atherogenesis. Mol Cell. (2001) 7:161–71. doi: 10.1016/s1097-2765(01)00164-2, 11172721

[101] Zhang Y Shen C Ai D Xie X Zhu Y. Upregulation of scavenger receptor BI by hepatic nuclear factor 4α through a peroxisome proliferator-activated receptor γ-dependent mechanism in liver. PPAR Res. (2011) 2011:164925. doi: 10.1155/2011/164925, 22190905 PMC3236442

[102] Vázquez-Carrera M Wahli W. PPARs as key mediators in the regulation of metabolism and inflammation. Int J Mol Sci. (2022) 23:025. doi: 10.3390/ijms23095025, 35563416 PMC9105541

[103] Hennig B Toborek M Joshi-Barve S Barger SW Barve S Mattson MP . Linoleic acid activates nuclear transcription factor-kappa B (NF-kappa B) and induces NF-kappa B-dependent transcription in cultured endothelial cells. Am J Clin Nutr. (1996) 63:322–8. 8602587 10.1093/ajcn/63.3.322

[104] Schuster S Johnson CD Hennebelle M Holtmann T Taha AY Kirpich IA . Oxidized linoleic acid metabolites induce liver mitochondrial dysfunction, apoptosis, and NLRP3 activation in mice. J Lipid Res. (2018) 59:1597–609. doi: 10.1194/jlr.M083741, 30084831 PMC6121934

[105] Zhang H Yu F Tian Z Jia D. Cardiolipin remodeling in cardiovascular diseases: implication for mitochondrial dysfunction. Acta Physiol (Oxford). (2025) 241:e70073. doi: 10.1111/apha.70073, 40530586

[106] Spiteller D Spiteller G. Oxidation of linoleic acid in low-density lipoprotein: an important event in Atherogenesis. Angew Chem Int Ed Eng. (2000) 39:585–9. doi: 10.1002/(SICI)1521-3773(20000204)39:3<585::AID-ANIE585>3.0.CO;2-G, 10671267

[107] Folcik VA Cathcart MK. Predominance of esterified hydroperoxy-linoleic acid in human monocyte-oxidized LDL. J Lipid Res. (1994) 35:1570–82. 7806971

[108] Poznyak AV Nikiforov NG Markin AM Kashirskikh DA Myasoedova VA Gerasimova EV . Overview of OxLDL and its impact on cardiovascular health: focus on atherosclerosis. Front Pharmacol. (2020) 11:613780. doi: 10.3389/fphar.2020.613780 33510639 PMC7836017

[109] Deol P Ruegger P Logan GD Shawki A Li J Mitchell JD . Diet high in linoleic acid dysregulates the intestinal endocannabinoid system and increases susceptibility to colitis in mice. Gut Microbes. (2023) 15:2229945. doi: 10.1080/19490976.2023.2229945, 37400966 PMC10321214

[110] Miyamoto J Igarashi M Watanabe K Karaki SI Mukouyama H Kishino S . Gut microbiota confers host resistance to obesity by metabolizing dietary polyunsaturated fatty acids. Nat Commun. (2019) 10:4007. doi: 10.1038/s41467-019-11978-0, 31488836 PMC6728375

[111] Qiu J Hu P Li F Huang Y Yang Y Sun F . Circulating linoleic acid and its interplay with gut microbiota during pregnancy for gestational diabetes mellitus. BMC Med. (2025) 23:245. doi: 10.1186/s12916-025-04061-7, 40289092 PMC12036143

